# Supplementary material for: CD44 Modulates Cell Migration and Invasion in Ewing Sarcoma Cells
Source: Int J Mol Sci. 2023 Jul 21;24(14):11774. doi: 10.3390/ijms241411774 (PMC10381016; doi:10.3390/ijms241411774)
Supplement: Supplementary file 1 [file ijms-24-11774-s001.zip › Supplementary_Figures_Fernandez-Tabanera.pdf]

# Supplementary Figures

## CD44 modulates cell migration and invasion in Ewing sarcoma cells

International Journal of Molecular Sciences

**Fernández-Tabanera et al.**
**Corresponding autor: Javier Alonso (fjalonso@isciii.es)**

**A**

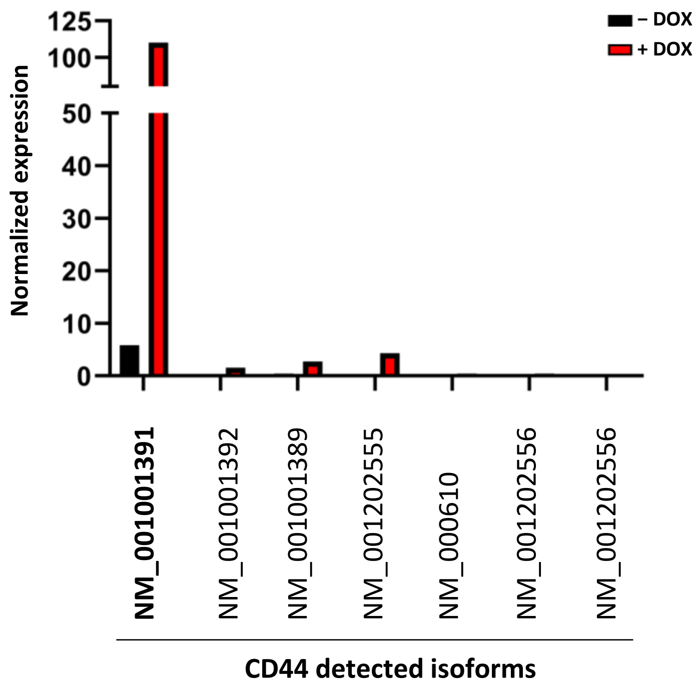


**Figure S1**. (A) Normalized expression of CD44 isoforms in the A673/TR/shEF RNAseq analysis. The most significantly overexpressed isoform, upon EWSR1:FLI1 knockout, was NM_001001391.

## Supplementary Figures Fernández-Tabanera et al.,

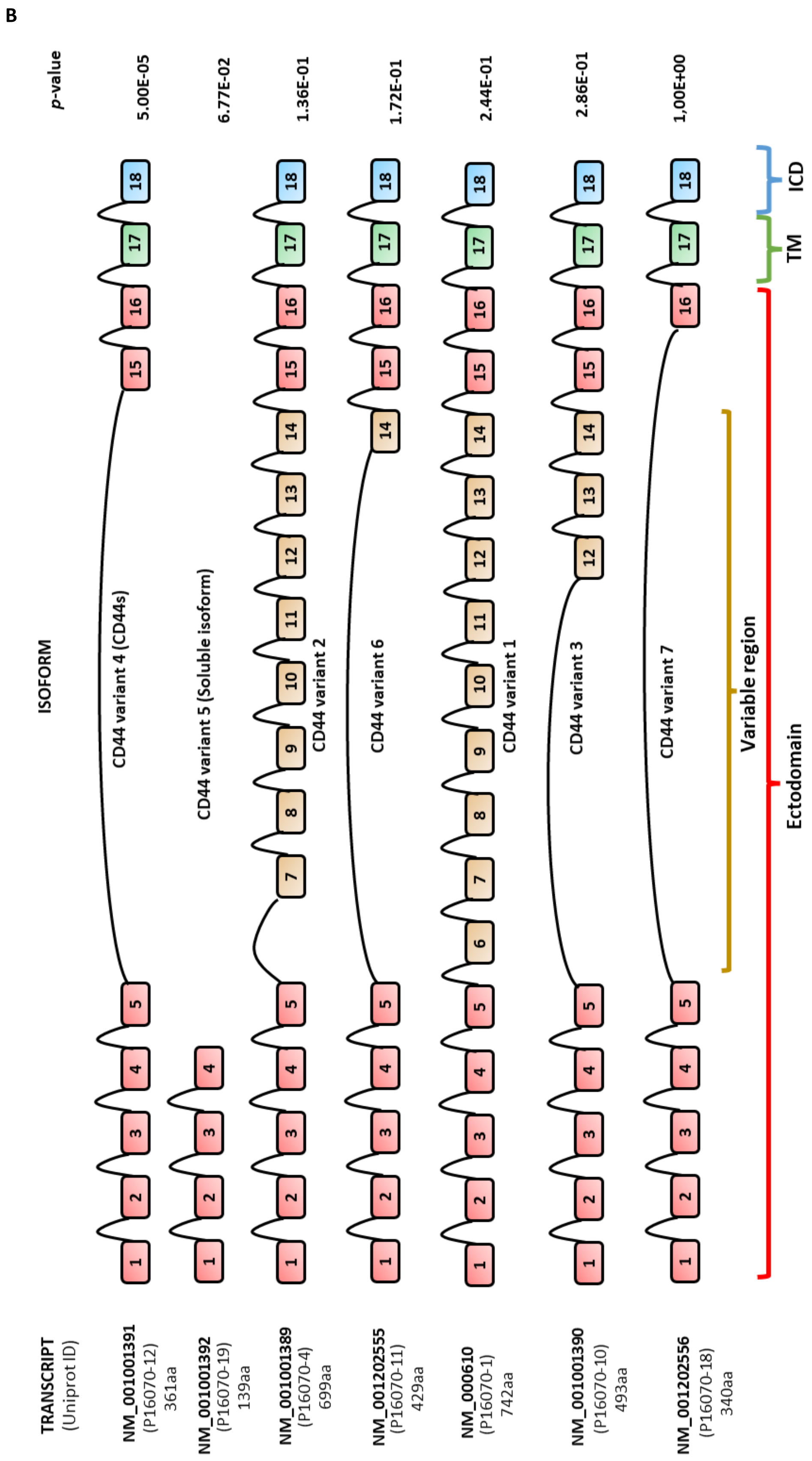


**Figure S1.** (B) Schematic representation of the CD44 isoforms detected in the A673/TR/shEF RNAseq analysis (including p-adjusted value). TM=transmembrane domain; ICD=intracellular domain

## Supplementary Figures Fernández-Tabanera et al.,

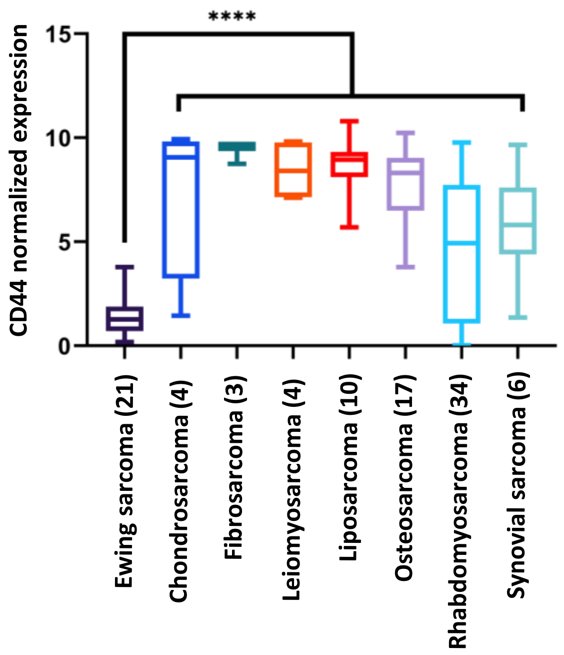


**Figure S2**. CD44 expression in several Ewing sarcoma (21), Chondrosarcoma (4), Fibrosarcoma (3), Leiomyosarcoma (4), Liposarcoma (10), Osteosarcoma (17), Rhabdomyosarcoma (34) and Synovial sarcoma (6) cell lines. Data extracted from DEPMAP portal (https://depmap.org/portal/). (median, 95%CI, from min to max value). ****p<0.0001; Student's t-test.

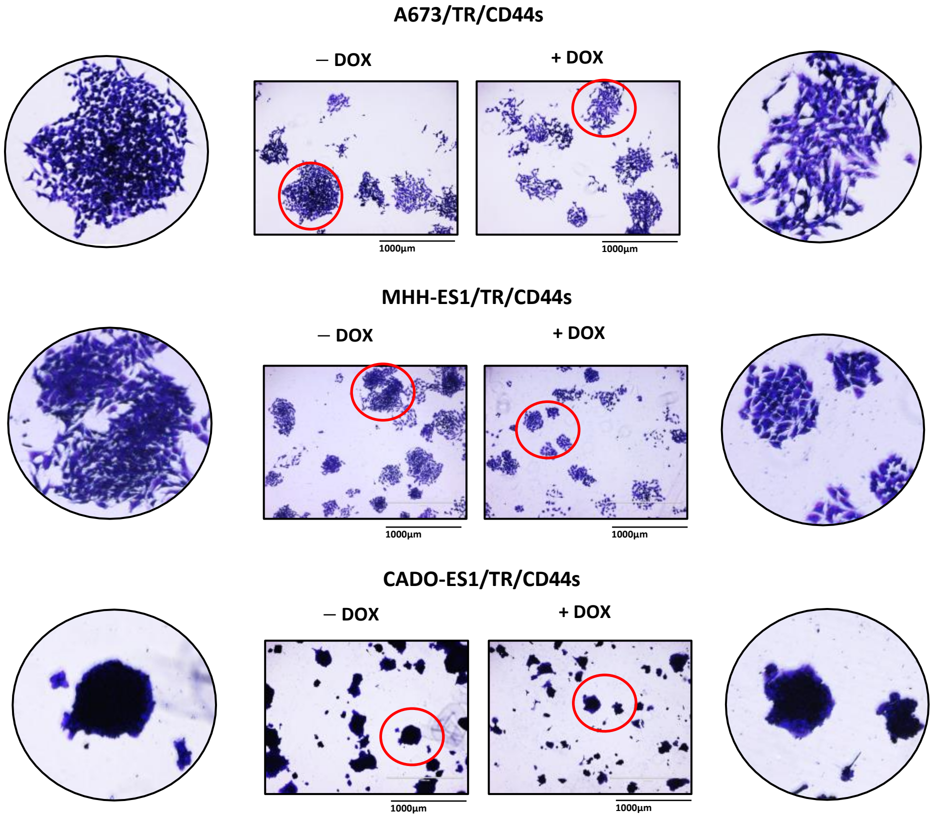


**Figure S3**. Representative images from the clonogenic assay in the three different TR/CD44s Ewing sarcoma cell lines.
